# *Pseudomonas aeruginosa* DnaK Stimulates the Production of Pentraxin 3 via TLR4-Dependent NF-κB and ERK Signaling Pathways

**DOI:** 10.3390/ijms22094652

**Published:** 2021-04-28

**Authors:** Jisu Jeon, Yeji Lee, Hyeonseung Yu, Unhwan Ha

**Affiliations:** 1 Department of Biotechnology and Bioinformatics, Korea University, Sejong 30019, Korea; allonso@korea.ac.kr (J.J.); yejee90@korea.ac.kr (Y.L.); cockychild@korea.ac.kr (H.Y.); 2 Interdisciplinary Graduate Program for Artificial Intelligence Smart Convergence Technology, Korea University, Sejong 30019, Korea

**Keywords:** DnaK, ERK, NF-κB, *Pseudomonas aeruginosa*, PTX3

## Abstract

Microbe-derived factors trigger innate immune responses through the production of inflammatory mediators, including pentraxin 3 (PTX3). PTX3 is a soluble pattern recognition molecule that stimulates the clearance of clinically important bacterial pathogens such as *Pseudomonas aeruginosa*. However, the *P. aeruginosa* factors responsible for the production of PTX3 have not been elucidated. In this study, we found that *P. aeruginosa* DnaK, a homolog of heat shock protein 70, induced PTX3 production. Induction was mediated by intracellular signals transmitted through the Toll-like receptor 4 (TLR4) signaling pathway. Following receptor engagement, the stimulatory signals were relayed initially through the nuclear factor kappa B (NF-κB) signaling pathway and subsequently by extracellular signal-regulated kinases (ERK), which are mitogen-activated protein kinases. However, ERK activation was negatively controlled by NF-κB, implying the existence of negative crosstalk between the NF-κB and the ERK pathways. These data suggest that *P. aeruginosa* DnaK acts as a pathogen-associated molecular pattern to trigger modulation of host defense responses via production of PTX3.

## 1. Introduction

Innate immune responses play essential roles in defending against microbial infections in animals (e.g., infections by avian influenza, swine influenza, *Aspergillus*, pathogenic *Escherichia coli* (*E. coli*), *Shigella*, and *Pseudomonas aeruginosa* (*P. aeruginosa*)) [1,2,3,4,5,6,7]. As critical players in innate immune responses, macrophages represent the first lines of host defense, which is modulated by the action of diverse pattern recognition molecules (PRMs). PRMs are critical for the recognition of conserved microbial moieties expressed on the surface or released by pathogens. They are divided into two groups based on their localization: cell-associated receptors (e.g., Toll-like receptors (TLRs), Nod-like receptors, and RIG-like receptors) and fluid-phase molecules (e.g., complement compartments, mannose-binding lectin, and pentraxins (PTXs)). In addition to their recognition effect, fluid-phase PRMs contribute to innate immunity by influencing the regulation of complement activation, opsonization of pathogens, and regulation of inflammation [8].

PTXs, which are soluble PRMs, are classified as long or short based on the length of the *N*-terminal region. C-reactive protein and serum amyloid P belong to the short family, whereas the long family includes PTX3, PTX4, and neuronal PTX [9]. PTX3 forms a 340 kDa octamer composed of two tetramers linked together by covalent bonds. This evolutionarily conserved structure allows for the recognition of diverse bacterial, fungal, and viral pathogens, such as *P. aeruginosa*, *Shigella flexneri* (*S. flexneri*), uropathogenic *E. coli*, *Aspergillus fumigatus*, and the influenza virus, in order to exert greater defensive activity [10,11,12,13,14,15]. Given that PTX3 plays a protective role in innate immune responses, it has been considered as a novel marker for infectious disease severity. Besides modulating the activity of innate immune mechanisms, PTX3 also plays essential roles in wound healing and tissue remodeling [8].

In response to diverse inflammatory stimuli, including TLR agonists and microbial moieties, PTX3 is expressed by a variety of stimuli and released by numerous cell types, such as dendritic cells, monocytes, macrophages, fibroblasts, epithelial cells, and vascular endothelial cells, to facilitate microbial clearance and protect the cell [8,16,17]. Previous work demonstrated the protective effects of PTX3 against respiratory infection with *Klebsiella pneumoniae* in transgenic mice that overexpress PTX3 [18]. In line with this, the physiological level of PTX3 was intentionally elevated by stimulating inflammatory signaling with lipopolysaccharide (LPS) following *Shigella* infection, proving that the elevation strongly correlates with the severity of shigellosis symptoms [12]. To extend the potential therapeutic application of PTX3, the infected animals were treated with recombinant PTX3 proteins, which led to improvements in recognition and phagocytic clearance of microbial pathogens (e.g., *A. fumigatus*, *S. flexneri*, and *P. aeruginosa*) [11,12,17,19,20]. These results imply that PTX3 proteins play roles in fighting against infection and highlight the potential use of PTX3 level as a biomarker for infection severity. 

In a previous report, we showed that PTX3 expression is stimulated in response to *P. aeruginosa* infection and that *P. aeruginosa*-derived GroEL is a released factor involved in upregulating the expression in human macrophages [21]. In this study, investigations aimed at identifying additional *Pseudomonas* factors revealed that *P. aeruginosa*-derived DnaK, a homolog of heat shock protein 70 (HSP70), contributes to the upregulation of PTX3 production. *P. aeruginosa* DnaK stimulated the expression of PTX3 via the TLR4-dependent nuclear factor-kappa B (NF-κB) and extracellular signal-regulated kinase (ERK) signaling pathways. Of note, DnaK-induced PTX3 expression was increased by the activity of NF-κB, followed by the activation of ERK, which is negatively regulated by NF-κB. Overall, our results suggest that DnaK has therapeutic potential: it could be used to trigger innate defense responses by increasing PTX3 production in response to microbial infections.

## 2. Results

### 2.1. P. aeruginosa-Derived DnaK Increases the Production of PTX3

**Figure 1:**
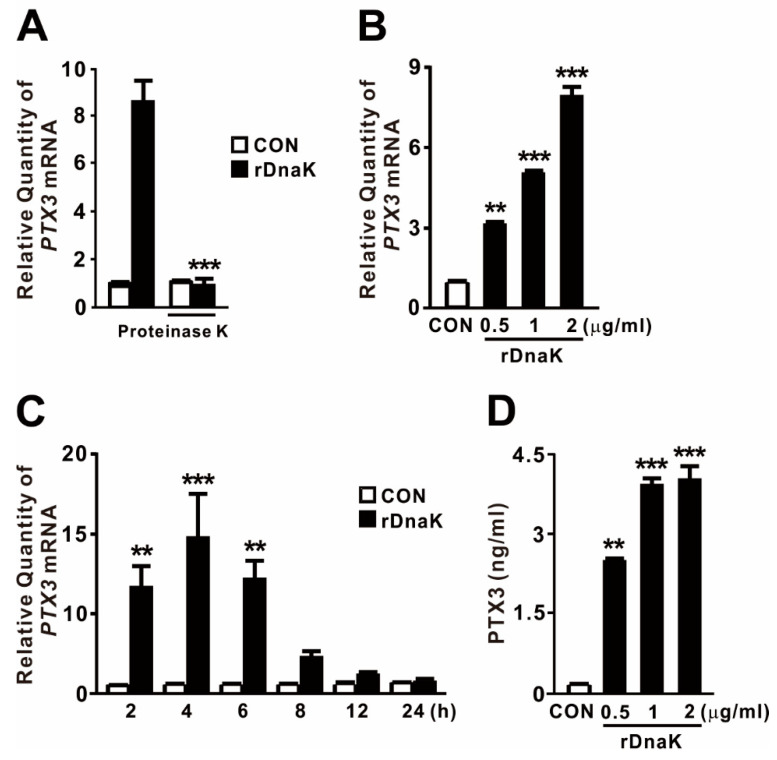
*P. aeruginosa*-derived DnaK increases the production of PTX3. (**A**) Recombinant *P. aeruginosa* DnaK (rDnaK) was pretreated with proteinase K (20 μg/mL) for 1 h, followed by the treatment of dTHP-1 cells with rDnaK (0.5 μg/mL) for 4 h. (**B**–**D**) The dTHP-1 cells were treated with the indicated concentrations of rDnaK for either 4 h (**B**) or 24 h (**D**), as well as with 0.5 μg/mL rDnaK for the indicated time (**C**). After treatment, the level of PTX3 expression was quantified by either qRT-PCR or ELISA. Data in (**A**–**D**) are expressed as mean ± SD (*n* = 3). ** *p* < 0.01; *** *p* < 0.001 vs. no treatment (**A**) or CON (**B**–**D**).

Eukaryotic HSP70 is a damage-associated molecular pattern (DAMP) that is strongly induced under various stresses, including infection, to protect the cell [22]. DnaK, a bacterial homolog of the HSP70 family, may act similarly by modulating innate immune responses through the induction of PTX3 expression. To examine the effect of DnaK on the expression of PTX3, we treated macrophages with *P. aeruginosa* DnaK. As shown in Figure 1A, expression of *PTX3* was markedly increased by treatment with recombinant DnaK (rDnaK). Notably, the application of proteinase K decreased the rDnaK-mediated expression of *PTX3*, implying that the induction was mediated by a protein factor. PTX3 expression was induced at the protein and mRNA levels in response to rDnaK in a dose-dependent manner (Figure 1B,D). Production reached a maximum level after 4 h of treatment and began to decrease thereafter (Figure 1C). Taken together, these results demonstrate that *P. aeruginosa* DnaK potently induces production of PTX3 in macrophages.

### 2.2. DnaK-Induced Production of PTX3 Is Mediated by the NF-κB and ERK Signaling Pathways

**Figure 2:**
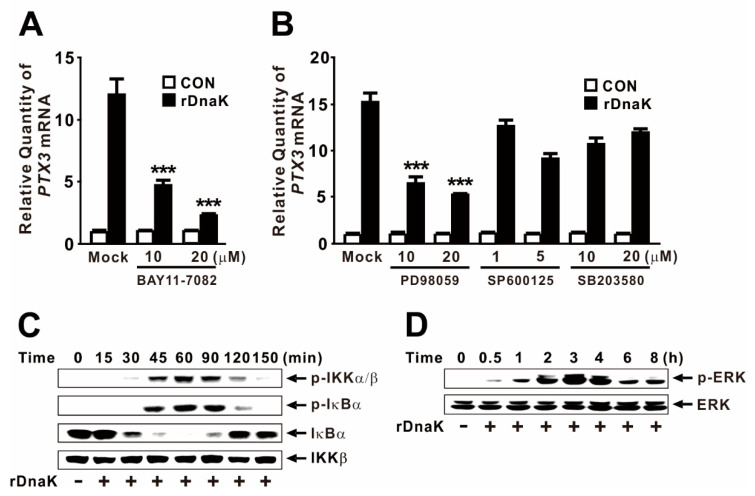
DnaK-induced production of PTX3 is mediated by the NF-κB and ERK signaling pathways. (**A**,**B**) The dTHP-1 cells were pretreated with the indicated concentrations of chemical inhibitors for 1 h followed by treatment with 1 μg/mL rDnaK for 4 h. (**C**,**D**) The dTHP-1 cells were treated with 1 μg/mL rDnaK for the indicated time. After treatment, qRT-PCR and immunoblot analyses were performed. Data in (**A**,**B**) are expressed as means ± SD (*n* = 3). Data in (**C**,**D**) are representative of three separate experiments. *** *p* < 0.001 vs. rDnaK treatment in the absence of inhibitors (**A**,**B**).

A regulatory region within the human *PTX3* promoter contains binding sites for transcription factors, including NF-κB and AP-1 [9,23]. To determine whether rDnaK induces the transcription of *PTX3* through these factors, we pretreated dTHP-1 cells with BAY 11-7082, a specific chemical inhibitor of IKK, to block NF-κB activity prior to rDnaK treatment. In addition, cells were pretreated with mitogen-activated protein kinase (MAPK) inhibitors, such as PD98059 (a specific chemical inhibitor of ERK), SP600125 (a specific chemical inhibitor of JNK), and SB203580 (a specific chemical inhibitor of p38). As shown in Figure 2A,B, *PTX3* expression was decreased by pretreatments with either BAY 11-7082 or PD98059, suggesting that NF-κB and ERK are involved in the DnaK-mediated induction of *PTX3* expression. Notably, we did not observe a significant change in expression with the pretreatment of other MAPK inhibitors. In this context, we found that the NF-κB signaling pathway was activated most strongly at 1 h in response to the rDnaK treatment, based on the phosphorylation of both IKKα/β and IκBα, and the degradation of IκBα (Figure 2C). Activation of the ERK signaling pathway was monitored by measuring phosphorylation of ERK, which was highest at around 3 h (Figure 2D). Taken together, these results indicate that rDnaK-mediated *PTX3* expression is under the control of the NF-κB and ERK signaling pathways.

### 2.3. ERK Engages in Negative Crosstalk with the NF-κB Signaling Pathway

**Figure 3:**
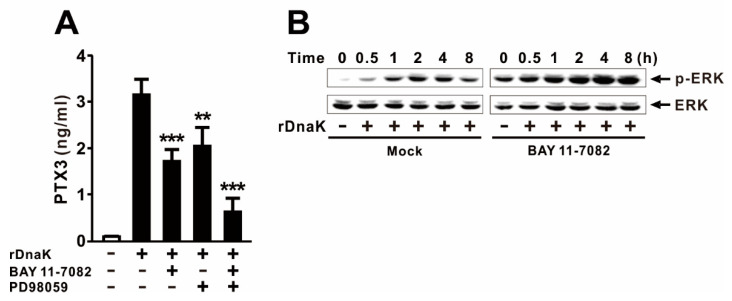
ERK engages in negative crosstalk with the NF-κB signaling pathway. The dTHP-1 cells were pretreated for 1 h with 10 μM of either BAY 11-7082 or PD98059 and then with 1 μg/mL rDnaK for either 24 h (**A**) or the indicated times (**B**). After treatment, ELISA and immunoblot analyses were performed. Data in (**A**) are expressed as means ± SD (*n* = 3). Data in (**B**) are representative of three separate experiments. ** *p* < 0.01; *** *p* < 0.001 vs. rDnaK treatment in the absence of inhibitors.

Upon elucidating the involvement of both signaling pathways, we examined the effects of treating the cell with both inhibitors. As shown in Figure 3A, production of PTX3 was decreased to a greater extent by pretreatment with both inhibitors than with either treatment alone, suggesting that NF-κB and ERK operate in parallel in the DnaK-mediated induction of PTX3 expression. Given that the effects of NF-κB and ERK were strongest at 1 and 3 h, respectively, we sought to characterize the crosstalk between the two signaling pathways. To determine the effect of activated NF-κB on the activation of ERK, we measured phosphorylation of ERK in the presence and absence of BAY 11-7082. The inhibitor increased ERK phosphorylation (Figure 3B), indicating that ERK activation was negatively controlled by NF-κB. These observations indicate that the rDnaK-mediated intercellular signal initially activates the NF-κB pathway to induce the expression of PTX3. Subsequently, reduced activation of NF-κB around 3 h allows ERK to be activated, continuing the stimulation of PTX3 production. 

### 2.4. DnaK-Induced Expression of PTX3 Is under the Control of TLR4

**Figure 4:**
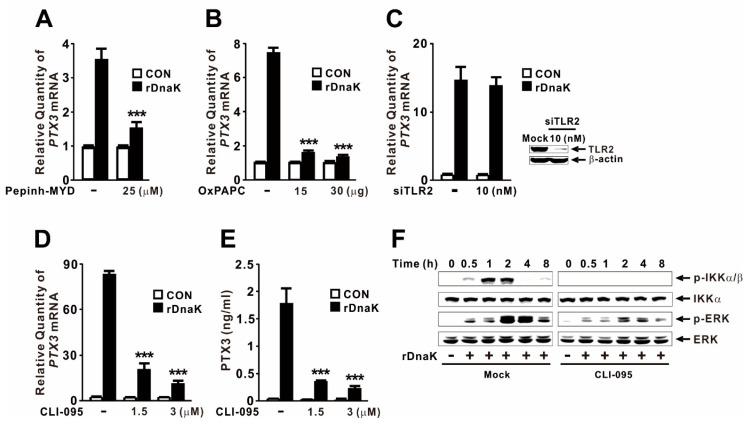
DnaK-induced expression of PTX3 is under the control of TLR4. The dTHP-1 cells were pretreated with either 25 μM Pepinh-MYD for 6 h (**A**), the indicated concentrations of OxPAPC for 1 h (**B**), the indicated concentrations of CLI-095 for 6 h (**D**,**E**), or 1.5 μM CLI-095 for 6 h (**F**), followed by treatment with 1 μg/mL rDnaK for 4 h (**A**,**B**,**D**), 24 h (**E**), or the indicated times (**F**). (**C**) The dTHP-1 cells were transfected with 10 nM TLR2 siRNA (siTLR2). Forty-eight hours post-transfection, the transfected cells were treated with rDnaK (1 μg/mL) for 4 h. After treatment, qRT-PCR, ELISA, and immunoblot analyses were performed. Data in (**A**–**E**) are expressed as means ± SD (*n* = 3). Data in (**F**) are representative of three separate experiments. *** *p* < 0.001 vs. rDnaK treatment in the absence of inhibitors (**A**,**B**,**D**,**E**).

TLRs are type I transmembrane receptors that detect conserved PAMPs and DAMPs, including HSPs [24]. To determine whether TLRs are involved in triggering PTX3 expression in response to DnaK, we pretreated macrophages with Pepinh-MYD, a chemical inhibitor of MyD88. As shown in Figure 4A, pretreatment decreased the expression of *PTX3*, indicating that induction is mediated by TLR signaling. Given that released HSP70 activates immune cells by engaging the TLR2 and TLR4 signaling pathways [25], we pretreated macrophages with OxPAPC, a chemical inhibitor of TLR2 and TLR4, to determine the effect of these two TLRs. Again, the inhibitor decreased *PTX3* expression (Figure 4B). To determine which TLR was responsible, we first investigated the effect of TLR2 in the rDnaK-mediated expression of *PTX3* by performing siRNA interference. As shown in Figure 4C, transfection with *TLR2* siRNA (siTLR2) did not cause any differences in expression. Knockdown by the siRNA was verified by immunoblotting for the TLR2 protein. We then applied CLI-095, a specific chemical inhibitor of TLR4, to macrophages, and observed a reduction in PTX3 expression at the mRNA and protein levels (Figure 4D,E). Finally, we investigated whether rDnaK-mediated activation of NF-κB and ERK is under the control of TLR4 by measuring the phosphorylation of IKKα/β and ERK in the presence and absence of CLI-095. As shown in Figure 4F, phosphorylation of both kinases was diminished by pretreatment with the inhibitor, suggesting that they are under the control of the TLR4 signaling pathway. Taken together, these observations indicate that rDnaK induces PTX3 expression in a TLR4-dependent manner.

## 3. Discussion

The innate immune system modulates host defense responses to *P. aeruginosa* by recognizing diverse bacterial factors either associated with or released from the pathogen during infection. Identification of microbial factors contributes to the understanding of these recognition events and the establishment of defense responses against *P. aeruginosa*. In addition, these factors could be used therapeutically to boost the fight against infection. HSPs are a family of chaperon proteins that are highly conserved in both prokaryotic and eukaryotic cells. Two major chaperone systems present in bacteria include GroEL (HSP60)-GroES (HSP10) and DnaK (HSP70)-DnaJ (HSP40)-GrpE (HSP20) [26]. Previously, we reported that increased expression of *PTX3* is mediated not only by *P. aeruginosa* infection, but also by treatment with proteins larger than 50 kDa present in the supernatant of *P. aeruginosa* cultures; this led to identification of the inductive effect of GroEL [21]. DnaK is also a protein larger than 50 kDa and was identified via proteome analysis as an extracellular protein released from *P. aeruginosa* along with GroEL [27]. This led us to investigate DnaK as an inducing factor for the production of PTX3.

The rDnaK protein was obtained under endotoxin-free purification conditions as described previously [28]. Because the recombinant protein was produced in *E. coli*, we were concerned that LPS might be carried over during the purification procedure, contributing to the induction of *PTX3*. To eliminate this possibility, we pretreated the purified rDnaK protein with Triton X-114, which eliminates most residual LPS present from purified proteins. The LAL endotoxin assay revealed that this pretreatment decreased the level of LPS contamination to < 0.5 ng/mL, which is not sufficient to induce *PTX3* expression under our experimental conditions. As shown in Figure 1B,D, pretreated rDnaK still clearly induced the expression of PTX3 in a dose-dependent manner, and treatment with proteinase K prevented induction (Figure 1A), suggesting that LPS does not play a role in the induction. Consistent with this, we previously demonstrated the effect of rDnaK protein on the expression of IL-1β and IL-27 in macrophages [28,29,30]. Thus, we concluded that *P. aeruginosa* DnaK is a potent inducing factor for expression of PTX3.

Eukaryotic HSPs are DAMPs that promote NF-κB activation and cytokine release by engaging signaling pattern recognition receptors such as TLRs in macrophages [31]. TLR4 deficiency impairs bacterial clearance by causing defects in the proinflammatory response and decreasing leukocyte recruitment in response to HSP60 [32]. In addition, the HSP70 family activates immune cells by binding to TLR4, promoting the production of inflammatory cytokines and leading to strong innate immune responses against infections [25,33,34,35]. We also observed that the *PTX3* expression was influenced by specific chemical inhibitors targeting MyD88 and TLR2/4 (Figure 4A,B), indicating that DnaK-mediated induction is also under the control of the TLR2 or TLR4 signaling pathways. Subsequently, we found that *P. aeruginosa*-derived DnaK is recognized not via TLR2, as determined using a loss of function approach (Figure 4C), but via TLR4, as determined using specific chemical inhibitors (Figure 4D,E). Consistent with this, rDnaK obtained from *Francisella tularensis* stimulates murine bone marrow-derived dendritic cells through TLR4 [36].

NF-κB and MAPK are evolutionarily conserved proteins that mediate signaling through cell membrane receptors such as TLRs to regulate intracellular targets. Upon recognition to TLR4, the binding of rDnaK transmits signals through the activation of NF-κB and ERK, providing important insight into the control of PTX3 expression in human macrophages (Figure 4F). However, the activation of NF-κB was strongest 1 h after treatment with rDnaK as demonstrated by measuring the phosphorylation levels of IKKα/β and IκBα as well as the degree of IκBα degradation (Figure 2C). By contrast, phosphorylation of ERK was highest after 3 h of post-treatment with rDnaK (Figure 2D). Of note, JNK and p38 MAPKs had no significant effect on PTX3 induction (Figure 2B). In addition, expression of PTX3 was decreased more by pretreatment with NF-κB and ERK inhibitors than by either inhibitor alone. These findings revealed a potential role for NF-κB as an upstream controller of ERK activation as verified by the induction of ERK phosphorylation in the presence of the NF-κB inhibitor (Figure 3B). This observation indicates that NF-κB inhibits the ERK pathway, consistent with previous reporting [37].

HSP70 family members are highly conserved from prokaryotes to higher eukaryotes in terms of their sequences and structures. Based on the high degree of sequence similarity (about 60%), we predicted that the bacterial HSP70 protein DnaK should have similar biological activity, especially with regard to protection against bacterial infection [38]. Consistent with this, treatment of monocytes with *E. coli*-derived DnaK increased the expressions of inflammatory cytokines, such as interleukin-6 (IL-6) and tumor necrosis factor α (TNFα) [39], suggesting a role for extracellular DnaK in the stimulation of immune responses. Furthermore, ingestion of *E. coli* overproducing DnaK significantly improved the survival of *Artemia* upon challenge with *Vibrio campbellii* [40]. Previously, we showed that soluble proteins in the culture supernatant from *P. aeruginosa* culture increased *PTX3* expression, ultimately revealing the role of GroEL in induction [21]. Consistent with this, in this study we demonstrated that rDnaK induces PTX3 expression by activating the NF-κB and ERK pathways in a TLR4-dependent manner. In humoral innate immunity, PTX3 plays an important role in preventing pulmonary infection by promoting *P. aeruginosa* clearance, demonstrating the therapeutic potential of PTX3 in *P. aeruginosa* lung infection [11]. These observations imply that microbial moieties such as DnaK could have therapeutic benefit by promoting immune modulation to control bacterial infections.

## 4. Materials and Methods

### 4.1. Reagents 

Proteinase K was purchased from Thermo Fisher Scientific (Waltham, MA, USA). Bay11-7082 and SP600125 were purchased from A.G. Scientific (San Diego, CA, USA). PD98059 and SB203580 were purchased from Selleck (Houston, TX, USA). Pepinh-MYD, OxPAPC, and CLI-095 were purchased from Invivogen (San Diego, CA, USA).

### 4.2. Cell Culture

THP-1 (human monocyte) cells were cultured in RPMI (Roswell Park Memorial Institute) 1640 (HyClone, Rockford, IL, USA). Culture media were supplemented with 10% heat-inactivated FBS (fetal bovine serum, HyClone) and antibiotics (100 units/mL penicillin and 0.1 mg/mL streptomycin). Differentiation of THP-1 was stimulated by treatment with 100 ng/mL PMA (phorbol-12-myristate-13-acetate) for 16 h and the resultant cells were designated as dTHP-1. Mammalian cells were cultivated at 37 °C in a humidified incubator under an atmosphere containing 5% CO_2_.

### 4.3. Construction and Purification of Recombinant DnaK Protein

The rDnaK protein was constructed and purified as described in our previous reports [28,29]. Phase-separation treatment with Triton X-114 was applied to remove endotoxin as described previously [41,42]. Endotoxin concentration was < 0.5 EU/mL protein as determined using the Limulus Amebocyte Lysate (LAL) Chromogenic Endotoxin Quantitation Kit (Pierce Thermo, Rockford, IL, USA). To obtain a control extract, *E. coli* strain BL21 (DE3) harboring the empty vector pETDuet-1 was subjected to the same procedures. The control extract was used to evaluate the effect of rDnaK throughout the study.

### 4.4. Quantitative Real-Time PCR (qRT-PCR) for PTX3 mRNA

Total RNA was isolated using TRIzol reagent (Invitrogen, Grand Island, NY, USA). The cDNA was synthesized using the ReverTra Ace qRT-PCR kit (Toyobo, Osaka, Japan). The qRT-PCR was performed using the SYBR Green PCR master mix (Kapa Biosystems, Woburn, MA, USA). Primer sequences were as follows: *PTX3*, 5′-TTGGACAACGAAATAGACAATGGA-3′ and 5′-GTCGTCCGTGGCTTGCA-3′; *GAPDH*, 5′-CCCTCCAAAATCAAGTGG-3′ and 5′-CCATCCACAGTCTTCTGG-3′. The qRT-PCR reactions were processed on a CFX96 Real-Time PCR System (Bio-Rad, Hercules, CA, USA). Thermal conditions were as follows: stage 1, 50 °C for 2 min and 95 °C for 10 min; stage 2, 95 °C for 15 s and 60 °C for 1 min. Stage 2 was repeated for 40 cycles. The comparative CT method was used to calculate the relative level of *PTX3* mRNA after normalizing against the level of *GAPDH* in the same sample.

### 4.5. Immunoblotting Analysis

Cells were lysed on ice for 10 min in lysis solution (20 mM Tris-HCl (pH 7.4), 50 mM NaCl, 50 mM Na pyrophosphate, 30 mM NaF, 5 μM zinc chloride, 2 mM iodoacetic acid, and 1% Triton X-100 in distilled water) supplemented with 1 mM PMSF (phenylmethylsulfonyl fluoride; Thermo Fisher Scientific) and 0.1 mM sodium orthovanadate (Sigma-Aldrich, St. Louis, MO, USA). The lysates were collected by centrifugation at 10,000× *g* for 15 min at 4 °C, and protein concentration was determined by the BCA (bicinchoninic acid) method (Pierce). Total proteins were separated by 10% SDS-PAGE and transferred to 0.45 μm PVDF (polyvinylidene difluoride) membranes. After the membranes were blocked at room temperature for 1 h in TBS (10 mM Tris-HCl (pH 7.5), 150 mM NaCl) containing 5% nonfat dry milk, they were incubated for 16 h at 4 °C with primary antibodies specific for p-IκB kinase (p-IKK) α/β (16A6), IKKα, IKKβ, p-IκBα, IκBα, p-ERK, ERK, TLR2, or β-actin (Cell Signaling Technology, Danvers, MA, USA). After incubation with the primary antibody, the blots were washed and incubated with the appropriate secondary antibodies followed by visualizing with the SuperSignal™ West Femto Maximum Sensitivity Substrate (Thermo Fisher Scientific) on an ImageQuant LAS 4000 (GE Healthcare Life Sciences, Pittsburgh, PA, USA).

### 4.6. Enzyme-Linked Immunosorbent Assay (ELISA)

The amount of PTX3 protein released into supernatants was measured using the Human Pentraxin 3/TSG-14 Quantikine ELISA Kit (R&D Systems, Minneapolis, MN, USA).

### 4.7. Transfection of siRNA

Cells (9 × 10^5^/mL) cultured in 12-well culture plates were transfected with the recommended concentrations of siRNA targeting TLR2 (siTLR2, #sc-40256; Santa Cruz Biotechnology, Dallas, TX, USA) using Lipofectamine RNAi Max (Invitrogen). Transfected cells were cultivated in RPMI 1640 supplemented with 10% FBS for 48 h at 37 °C. Transfection efficiency was assessed using cells transfected with FAM-labeled mimics. Total RNA and protein were harvested for qRT-PCR and immunoblot analysis, respectively.

### 4.8. Statistical Analysis

Statistical analyses were performed using Student’s *t*-tests or one-way ANOVA analyses followed by Tukey’s post hoc multiple range tests using the Instat package from GraphPad v.3.06 (GraphPad Software, Inc., San Diego, CA, USA). The value of *p* < 0.05 was considered statistically significant.

## 5. Conclusions

Soluble forms of pattern recognition molecule PTX3 are critical for recognition of conserved molecular patterns present in pathogens, thereby building innate immunity against infection. As a molecular pattern released by *P. aeruginosa*, we found that DnaK, a homolog of HSP70, potently stimulates *PTX3* expression. In addition, we previously reported that GroEL, a homolog of HSP60, is released into the supernatant from *P. aeruginosa* cultures and increases *PTX3* expression. These imply the diverse effects of HSP homologs released from *P. aeruginosa*. In this study, the effects of DnaK are primarily associated with recognition by TLR4 and subsequent signal transduction via NF-κB and ERK pathways in human macrophages. Such studies can contribute to our understanding of the diseases caused by *P. aeruginosa* infection and provide therapeutic opportunities for the control of bacterial infections.
